# A Dual-Chamber Serial–Parallel Piezoelectric Pump with an Integrated Sensor for Flow Rate Measurement

**DOI:** 10.3390/s19061447

**Published:** 2019-03-25

**Authors:** Song Chen, Mai Yu, Junwu Kan, Jianping Li, Zhonghua Zhang, Xinyi Xie, Xiaomin Wang

**Affiliations:** Institute of Precision Machinery and Smart Structure, Zhejiang Normal University, Jinhua 321004, China; chensong@zjnu.edu.cn (S.C.); zsdyumai@zjnu.edu.cn (M.Y.); lijp@zjnu.cn (J.L.); zhangzhh@zjnu.cn (Z.Z.); m17857091933@163.com (X.X.); 18395947619@163.com (X.W.)

**Keywords:** piezoelectric pump, integrated sensor, serial connection, parallel connection

## Abstract

A new concept of a dual-chamber serial-parallel piezoelectric pump with an integrated sensor (DSPPIS) is presented in this paper. By means of dividing a piezoelectric bimorph into an actuator and a sensor, sensing function is integrated onto the DSPPIS for flow rate measurement. A prototype of the DSPPIS was manufactured and assembled from a finished piezoelectric bimorph. Then, frequency and voltage characteristics were tested to evaluate the performance of the DSPPIS with serial and parallel connection. Experimental results show that the optimal frequency range of DSPPIS can be achieved and determined by itself through monitoring the sensing voltage when driven by a fixed voltage of 150 Vpp and a frequency range of 40–400 Hz. For a fixed frequency of 100 Hz and a voltage range of 50–250 Vpp, both the sensing voltage and output flow rate increase with the increase of driving voltage. It is observed that there is a positive correlation between sensing voltage and output flow rate, which was further fitted by using linear function. The correlation coefficients for the DSPPIS with serial and parallel connection are calculated as 0.9716 and 0.9054, respectively. As a result, the DSPPIS demonstrated in this paper has realized the measurement of flow rate without the additional flow-sensing equipment both in serial and parallel connection.

## 1. Introduction

Owing to the advantages of simple structure, small size, low power consumption and no electromagnetic noise, the piezoelectric pump has emerged as a critical research area for medicine, precision machinery, and biochemistry [1,2,3,4]. Particularly, to achieve high output performance, the piezoelectric pump with multi-chamber has been proposed [5,6,7]. Kan et al. designed and fabricated a multi-chamber piezoelectric micropump in serial connection, which achieved maximum pressure and flow rate of 48.6 kPa and 7.6 mL/min at a low driving voltage of 40 V, respectively [5]. Hwang et al. proposed two-chamber and three-chamber piezoelectric peristaltic pumps and the three-chamber piezoelectric pump achieved a higher flow rate than the pump with two-chamber under four working phases [6]. Zhang et al. presented a quintuple-chamber piezoelectric pump, the maximum flow rate of which is 67.8% higher than the pump with double working actuators [7]. 

In recent years, the dual-chamber single–actuator piezoelectric pump has attracted much attention for its high energy conversion efficiency [8,9]. Li et al. presented a valveless dual-chamber single–actuator piezoelectric pump, which achieved 1.3 times as much output flow rate compared with the pump with single chamber [8]. Furthermore, Zeng et al. developed a kind of dual-chamber single–actuator piezoelectric pump driven by a bimorph, which has 2 times as much output power compared with the pump with single chamber [9]. The aforementioned studies show that the use of series and parallel connection of dual-chamber could obviously improve the output flow and pressure. Each mode of dual-chamber connection has its advantages and disadvantages, which are suitable for different applications. We propose a dual-chamber serial-parallel piezoelectric pump (DSPP), which realizes the switching of series and parallel connection of dual-chamber single-actuator piezoelectric pumps by controlling the fluid passage, thereby obtaining the comprehensive advantages of serial and parallel connection and meeting the requirements of different applications. Despite above-excellent fluidic performances, the need for a monitoring of the output flow in serial and parallel connection remains a major issue for DSPP to have a feedback control on the delivery, or to detect malfunctions like occlusions or leakages.

To monitor flow rate, various pumps with integrated flow sensors have been developed [10,11,12,13,14]. Nguyen et al. integrated a micro mechanical flow sensor into a flexural-plate wave pump so that the pump was able to detect the slow velocity (<0.8 mm/s) of the acoustic streaming [10]. Zhang et al. presented a self-bended micro flow sensor fabricated from insulator wafers, providing the advantage of high sensitivity (1.5–3.5 Ω/cm/s) for small flow measurements (0–23 cm/s) [13]. Fuchs et al. presented a piezoelectric peristaltic pump with an integrated sensor which realized the flow rate sensing by fixing the silicon film directly on the sensor [12]. Zhang et al. put forward a piezoelectric pump with an integrated sensor by means of space-division multiplexing [14]. These works show that the integrated sensor can improve the performance of pump. Moreover, the integrated sensor can obtain more information than the external sensor, such as the deformation of vibrators and the pressure in pump chamber. However, most of the work was directed to single-chamber flow measurements, while flow measurements for dual-chamber in serial and parallel connection under the same structure were rarely reported.

A dual-chamber serial-parallel piezoelectric pump with an integrated sensor (DSPPIS) for flow rate measurement is developed in this paper to achieve output performance self-monitoring of a dual-chamber in serial and parallel connection, which will greatly improve the environmental adaptability and performance reliability of DSPP. The flow rate measurement method proposed in this paper realizes the driving and sensing function by means of dividing piezoelectric bimorph into an actuator and a sensor. The fluid operating state and sensing signal will change with the switching of serial and parallel connection. Through the study of sensing signals, the self-discrimination of serial/parallel connection and flow rate self-monitoring can be realized, which will lay a solid foundation for DSPPIS to achieve performance self-adaptation and serial/parallel connection self-switching in the future. DSPPIS is designed and manufactured in this work. Experimental analysis on the frequency and voltage characterization of DSPPIS under serial and parallel connection are explored.

## 2. Principle and Design 

**Figure 1:**
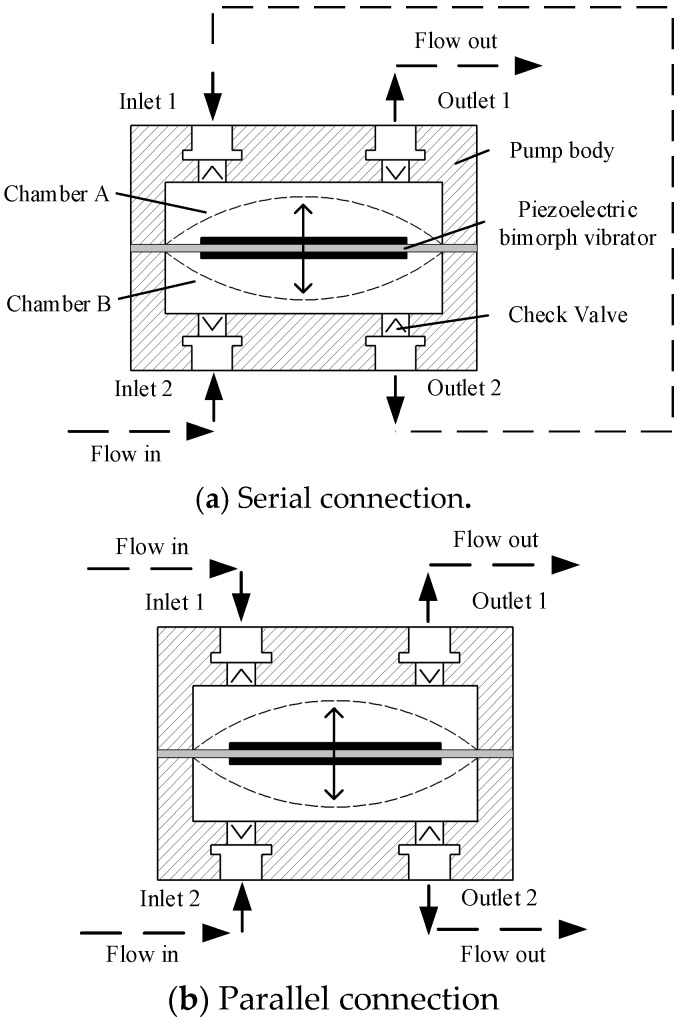
Schematic cross-section and connection mode of a dual-chamber serial-parallel piezoelectric pump with an integrated sensor (DSPPIS).

Figure 1 is the schematic cross-section and connection mode of DSPPIS, where the piezoelectric pump consists of piezoelectric bimorph vibrator, two chambers (chamber A and B), check valve, and pump body. Each chamber corresponds to a set of inlet (inlet 1 and inlet 2) and outlet (outlet 1 and outlet 2). With a serial connection that inlet 1 is connected to outlet 2, the fluid firstly flows into inlet 2 and lastly out at outlet 1. While in parallel connection, fluid flows into inlet 1 and 2 and out at outlet 1 and 2. 

**Figure 2:**
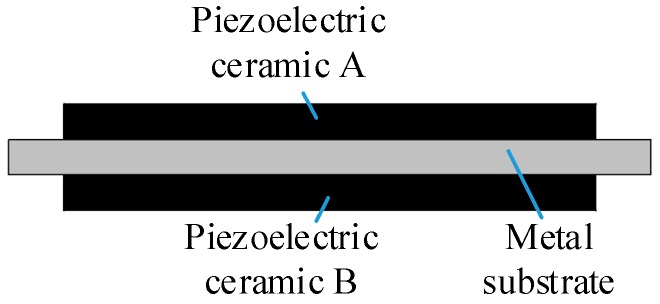
Schematic of the piezoelectric bimorph vibrator.

The piezoelectric bimorph vibrator is bonded by metal substrate, piezoelectric ceramic A (in chamber A) and piezoelectric ceramic B (in chamber B), as shown in Figure 2. In this paper, we take ceramic A as an actuator and ceramic B as a sensor. Driven by alternating current signals, the ceramic A vibrates and the ceramic B is forced to vibrate with the metal substrate. Due to the vibration, the ceramic B will generate corresponding voltage signals associated with vibration deformation under the action of positive piezoelectric effect. As the piezoelectric pump is a sort of cubage pump, there is a corresponding between deformation of the actuator unit with the output flow rate.

As the driving frequency (less than 400 Hz) is much lower than the natural frequency of the piezoelectric vibrator (more than 2.5 kHz), deformation of the center of circular piezoelectric vibrator is Refs. [14,15]:

(1) $$\delta =\frac{3d_{31}d^{2}U}{8t^{2}},$$

where $d_{31}$ is the piezoelectric constant; d is the diameter of piezoelectric membrane; t is the thickness of piezoelectric membrane; and U the is driving voltage. The sensing voltage of the piezoelectric ceramic acted as sensor is Ref. [2]:

(2) $$U_{s}=h_{31}\delta ,$$

where $h_{31}$ is the piezoelectric stiffness coefficient. Combining Equation (1) with Equation (2), the relationship between sensing voltage and driving voltage is:

(3) $$U_{s}=\frac{3h_{31}d_{31}d^{2}U}{8t^{2}}.$$

According to Equation (3), when the material, structure, and installation of the piezoelectric vibrator are fixed, the sensing voltage $U_{s}$ is in direct proportion to the driving voltage U. When the output pressure is zero, provided that the valve can be fully opened and closed, the output flow rate of the piezoelectric pump with single chamber and the single vibrator can be expressed as Ref. [16]:

(4) $$Q_{0}=\Delta Vf=\frac{\pi d^{2}}{8}\delta f,$$

where f is the driving frequency. For simplifying analysis, the performance of the two chambers is supposed to be identical. When two chambers are connected in serial, the output flow rate of the pump with two chambers is Refs. [5,7]:

(5) $$Q_{s}=\sqrt{2}Q_{0}.$$

Once two chambers are connected in parallel, obviously, each chamber has a separate inlet and outlet. The output flow rate of the pump with two chambers is:

(6) $$Q_{p}=2Q_{0}.$$

Thus, the relationship between sensing voltage and the output flow rate in serial and parallel connection can be written as:

(7) $$Q_{s}=\frac{\sqrt{2}\pi d^{2}f}{8h_{31}}\eta U_{s},$$

(8) $$Q_{p}=\frac{\pi d^{2}f}{4h_{31}}\eta U_{s},$$

where η is the efficiency parameter determined by the out-sync action of the piezoelectric vibrator and the valve. According to Equations (7) and (8), with the driving parameters and the structure of piezoelectric pump being determined, the sensing voltage is in direct correspondence with the output flow rate in serial and parallel connection. Accordingly, the self-sensing of flow rate can be realized by sensing voltage. Note that this case, the establishment of Equations (7) and (8) is based on the condition that driving frequency f (less than 400 Hz) is far away from the natural frequency $f_{0}$ (more than 2.5 kHz), so the driving voltage and frequency of the pump will affect efficiency parameter η.

## 3. Experimental Setup

**Figure 3:**
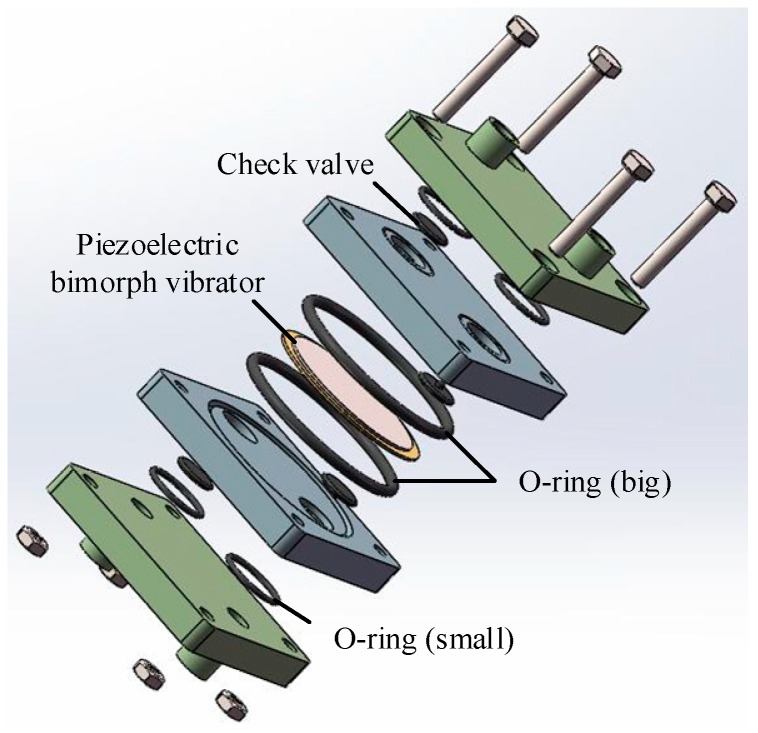
The 3-D assembly schematic of the DSPPIS.

The pump body is made of transparent polymethyl methacrylate (PMMA)to facilitate the observation of the pump chamber during the test. The valve is a kind of umbrella type and the material is silica gel. To fabricate the pump, we used bolts and an O-ring to connect and seal. Table 1 contains the structural parameters of DSPPIS. Figure 3 shows the 3-D assembly schematic of DSPPIS. 

**Figure 4:**
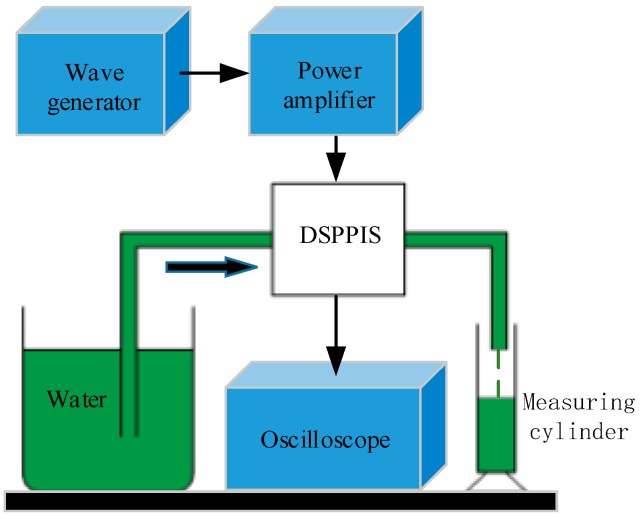
The schematic of the self-sensing test of the DSPPIS.

Figure 4 is the schematic of self-sensing test of the DSPPIS. The wave generator (Rigol, DG4162, Beijing, China) and power amplifier (Tabor, 9400, Tel Hanan, Israel) provide the driving signal of the sine wave for DSPPIS and the oscilloscope (GW instek, GDS-1102, Taipei, Taiwan) is used to collect the sensing voltage signal. The fluid medium is distilled water and output water flow rate is measured by a measuring cylinder. Initially, the output flow rate and sensing voltage are measured at the fixed driving voltage (150 Vpp) in serial and parallel connection. The fixed voltage of 150 Vpp can provide enough power as well as bandwidth. After that, the output flow rate and the sensing voltage are measured at the fixed frequency (100 Hz) in serial and parallel connection. The frequency of 100 Hz is far from the optimum working frequency of pump system so that the influence of system resonance can be reduced.

## 4. Experimental Results and Discussion

**Figure 5:**
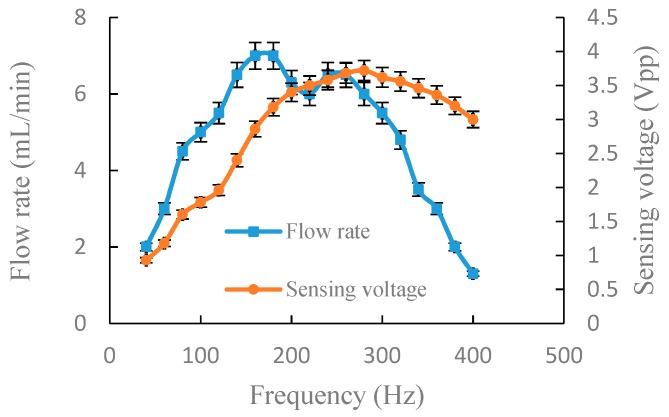
Output flow rate and sensing voltage under a fixed voltage of 150 Vpp in serial connection.

**Figure 6:**
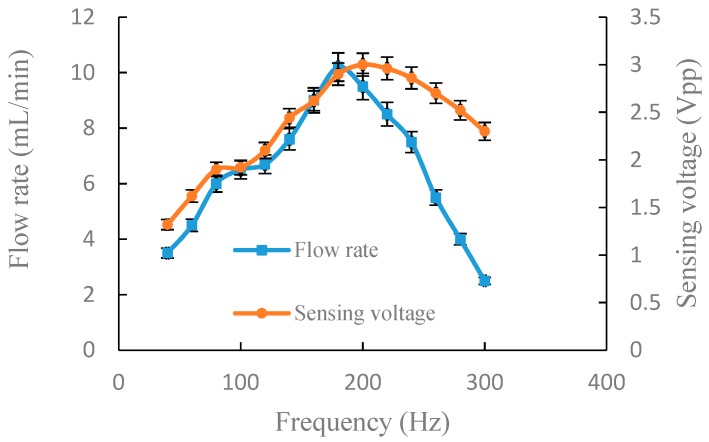
Output flow rate and sensing voltage under a fixed voltage of 150 Vpp in parallel connection.

**Figure 7:**
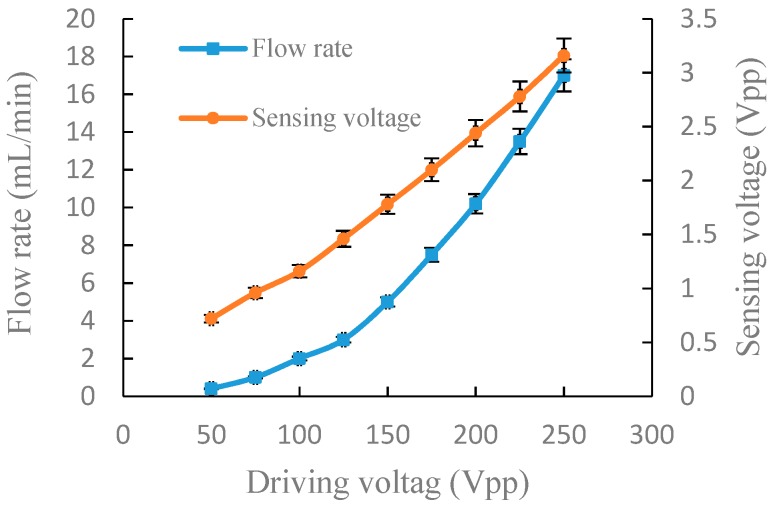
Output flow rate and sensing voltage under a fixed frequency of 100 Hz in serial connection.

**Figure 8:**
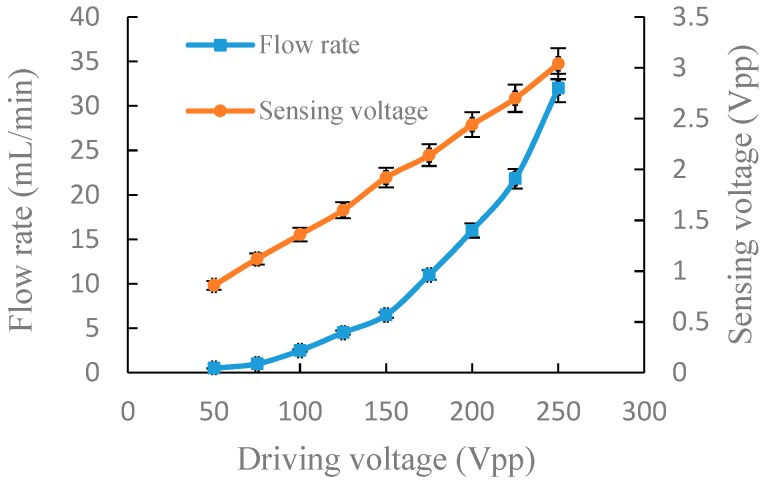
Output flow rate and sensing voltage under a fixed frequency of 100 Hz in parallel connection.

For the accuracy and authenticity of the data, we repeated the following experiment three times for each group. The relative standard deviation of the measured data was calculated and is indicated as error bars in Figure 5, Figure 6, Figure 7 and Figure 8.

Figure 5 shows the experimental results of the output flow rate and the sensing voltage under a fixed voltage of 150 Vpp in serial connection. When the driving frequency was 40–180 Hz, the output flow rate and sensing voltage increased with the increase of driving frequency. At 180 Hz, the output flow rate reached the primary peak (7.0 mL/min) and the sensing voltage was 3.18 Vpp. At 260 Hz, the output flow rate reached the secondary peak (6.5 mL/min) and the sensing voltage was 3.68 Vpp. Due to the deviation of the optimum working frequency of chamber A and B, the sensing voltage showed a rise at 180–280 Hz, but the output flow fluctuated slightly. However, the output flow changing trend was reflected by sensing voltage. After 280 Hz, the output flow rate and the sensing voltage decreased. Experimental results of Figure 5 indicate that the range of optimum frequency is 180–280 Hz and the sensing voltage reached maximum of 3.72 Vpp at 280 Hz. As a result, the frequency, which was less than and close to the frequency of maximum sensing voltage, could be selected as the driving frequency so that we were able to achieve a optimum output flow. For instance, 260 Hz was selected as driving frequency, so the output flow rate reached 6.5 mL/min, which was only 0.5 mL/min less than the maximum flow rate.

Keeping other parameters consistent, except the connection mode, Figure 6 illustrates the experiment results of output flow rate and sensing voltage in parallel connection. When the driving frequency was 40–180 Hz, the output flow rate and sensing voltage increased with the increase of the driving frequency. At 200 Hz, the sensing voltage reached maximum (3 Vpp) and the output flow rate was 9.5 mL/min. The range of optimum frequency was 180–200 Hz. After 200 Hz, the sensing voltage and output flow rate decreased with the increase of the driving frequency. Experimental results of Figure 6 indicate that there is a good positive correlation between output flow rate and sensing voltage. From the experimental results of Figure 5 and Figure 6, it can be known that the range of optimum frequency can be obtained through monitoring sensing voltage.

Figure 7 shows the experimental results of the output flow rate and the sensing voltage under a fixed frequency of 100 Hz in serial connection. The output flow rate and sensing voltage increased with the increase of the driving voltage. When the driving voltage was 50 Vpp, the output flow rate and sensing voltage were 0.4 mL/min and 0.72 Vpp, respectively. When the driving voltage increased to 250 Vpp, the output flow rate and sensing voltage increased to 17 mL/min and 3.16 Vpp, respectively.

Figure 8 shows the experiment results of the output flow rate and the sensing voltage under a fixed frequency of 100 Hz in parallel connection. Similarly, the output flow rate and sensing voltage increased with the increase of driving voltage. When the driving voltage was 50 Vpp, the output flow rate and sensing voltage were 0.5 mL/min and 0.86 Vpp, respectively. When the driving voltage increased to 250 Vpp, the output flow rate and sensing voltage increased to 32 mL/min and 3.04 Vpp respectively. 

**Figure 9:**
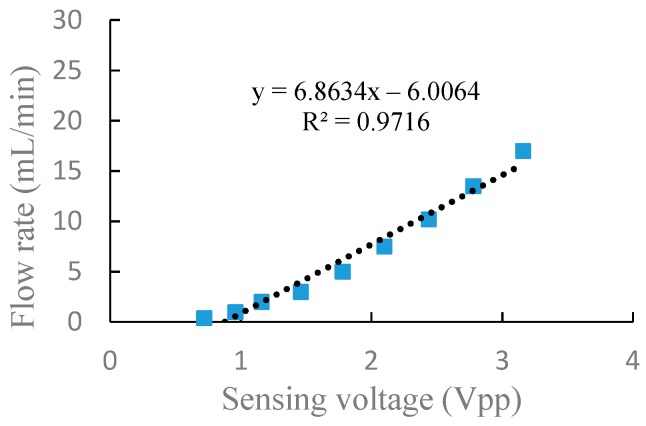
The relationship between flow rate and sensing voltage at 100 Hz in serial connection.

**Figure 10:**
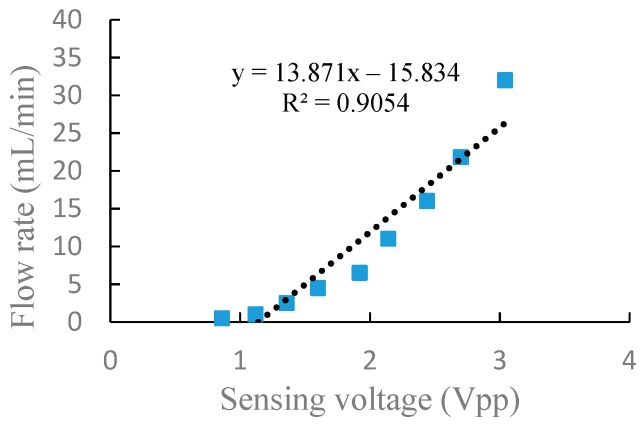
The relationship between flow rate and sensing voltage at 100 Hz in parallel connection.

Through the above experiments on frequency characteristics and voltage characteristics of the piezoelectric pump in serial and parallel connection, respectively, it can be seen from the experiment results that there is a corresponding relationship between the flow rate and the sensing voltage. In general, a piezoelectric pump is used to adjust the drive voltage at a fixed frequency to obtain the actual flow rate required. In order to better express the relationship between the flow rate and the sensing voltage at different driving voltage, a linear fitting performed by a least-square method was utilized to fit the flow rate and sensing voltage, as shown in Figure 9 and Figure 10. The correlation coefficients, $R^{2}$, are, respectively, 0.9716 and 0.9054 in serial and parallel connection, which shows that the flow rate of the DSPPIS in serial and parallel connection can be well reflected by sensing voltage. The increase of flow rate occurs more quickly in parallel than in serial connection with the increase of sensing voltage. Therefore, the sensing voltage signal is able to distinguish the different connection modes (serial and parallel connection).

## 5. Conclusions 

A dual-chamber serial–parallel piezoelectric pump with an integrated sensor (DSPPIS) was presented in this paper. The relationship between the sensing voltage and the output flow rate both in serial and parallel connection were obtained by experiments under different driving frequencies and voltages. The experimental results have shown that: (1) When the pump is driven by a fixed voltage of 150 Vpp and a frequency range of 40–400 Hz, the experimental results indicated that the sensing voltage reflects the changing trend of flow rate. Therefore, the optimal frequency range of DSPPIS can be achieved by monitoring the sensing voltage. (2) When the pump is driven by a fixed frequency of 100 Hz and a voltage range of 50–250 Vpp, the output flow rate corresponds well with the sensing voltage. Linear fitting performed by a least-square method was used to fit the data of flow rate and sensing voltage; the correlation coefficients, $R^{2}$, are 0.9716 and 0.9054 in serial and parallel connection, respectively. The increase of flow rate occurs more quickly in parallel than in serial connection with the increase of sensing voltage. Therefore, the sensing voltage signal is able to distinguish the different connection modes (serial and parallel connection).

## Figures and Tables

**Table 1:** Parameters of DSPPIS.

Type	Value and Material
Pump size	41 mm × 41 mm × 25 mm
Pump material	PMMA
Check valve type	Umbrella valve
Check valve material	Silica gel
Diameter of metal substrate	35 mm
Thickness of metal substrate	0.4 mm
Diameter of ceramic	28 mm
Thickness of ceramic	0.2 mm
Height of chamber	0.15 mm
Driving waveform	Sine wave
